# Remarks on the Causes and Cure of Some Diseases of Infancy

**Published:** 1800

**Authors:** Joseph Clarke

**Affiliations:** Licentiate in Physic of the Royal College of Physicians in Dublin, and M.R.I.A.


XXII. *Remarks on the Caufes and Cure of fome Difeafes of Infancy. By* Jofeph Clarke, *M. D. Licentiate in Phyfic of the Royal College of Phyficians in Dublin, and M. R. I. A. From the Tranfactions of the Royal Irifh Academy.* Vol. VI.

IT is now near feven years fince an effay of mine was read before this Academy, on the properties of human milk, the changes it undergoes in digeftion, and the difeafes fuppofed to originate from this fource in infancy. A variety of facts and obfervations was then brought forward to render it probable that the caufes, commonly alledged by writers to pro-

 duce duce moſt of the diſeaſes of infants, are ill-founded, nay, do not exiſt; and conſequently that the remedies propoſed for their cure muſt often prove ineffectual. Since the above period, my attention has been very much directed to this ſubject, and it is well known that my opportunities of experience have not been inconſiderable; and yet I every day feel more forcibly the evidence in favour of my former doubts. Once more, therefore, I am tempted to ſolicit attention to this ſubject, by ſubmitting the following remarks, however curſory and imperfect, to public conſideration: They relate principally to four diſeaſes, viz.

I. Diarrhœa, accompanied with much griping and green ſtools.

II. Obſtinate coſtiveneſs.

III. Nine day fits, or convulſions in early infancy.

IV. Cutaneous eruptions.

As it can be of no uſe to repeat what preceding writers have ſaid on theſe ſubjects, I ſhall confine myſelf to ſuch remarks as are not commonly to be met with in print.

In the eſſay above mentioned* I endeavoured

* See Tranſactions of the Royal Iriſh Academy, for the year 1788, and London Medical Journal, Vol. XI.

to to prove that green ſtools in infancy are not to be conſidered as ſure ſigns of the exiſtence of a ſuperabundant acid in the ſtomach and inteſtines; at that time I was totally ignorant of their nature. An accident firſt ſuggeſted to me new ideas on this ſubject. A lady of very acute talents took it into her head that the milk of a nurſe in her ſervice did not agree with her infant. One night this infant, after ſuffering a great deal of griping, paſſed a remarkably green ſtool, which the mother conſidered as a deciſive proof that nurſe's milk turned ſour in her child's ſtomach, and of courſe muſt diſagree with it. The cloth on which this ſtool was received happened to be thrown into a corner of the room, where it lay till next morning, when the nurſe, to her great ſurprize, found it had changed the *green* for a *natural yellow* colour. The miſtreſs ſuſpected miſrepreſentation on the part of the nurſe, and I was ſent for to decide between them. Not knowing well how to give a ſatisfactory determination, I begged of the lady to ſuſpend her judgment, and to remark particularly whether in future ſuch changes, as the nurſe deſcribed, would happen. She made the experiment repeatedly with great care, and uniformly found that that the green colour changed to a yellow by time, and perhaps by being diffufed on the cloth. A very particular attention to this cafe led me ftrongly to fufpect that green ftools in infancy (which medical writers have long imputed to predominant acidity) are often *really* of a *bilious* nature. The inefficacy of abforbent medicines to correct them, joined to fome other confiderations to be hereafter noticed, ferved ftill farther to confirm my fufpicions. Impreffed ftrongly with thefe ideas, I determined to deviate from common practice, by having recourfe to the ufe of calomel, one of the few remedies which experience has found powerfully to influence the biliary fecretion in adults. It anfwered my expectation fo well then, and in many fimilar inftances fince, that I am perfuaded it may be of fome utility briefly to ftate the refult of my obfervations refpecting it.

As very green ftools are generally preceded by and accompanied with a great deal of griping and diftrefs to the infant, they feem to me to indicate unufual acrimony in the bile, and probably fome degree of depraved fecretion in the liver. Sometimes this morbid tendency is of fhort duration, fometimes it continues troublefome troublefome for weeks. In the former cafes, caftor oil in moderate quantity will be found a good remedy; it evacuates fpeedily the contents of the bowels, and at the fame time fheaths the infide of the inteftinal canal againft their acrimony. In the more obftinate cafes, where oil only affords temporary relief, calomel is the only remedy I have found produce any permanent good effects. I am inclined to think it operates not merely by evacuating, but by correcting that tendency to depraved fecretion, which in bad cafes probably exifts. To infants under fix months old I generally begin with half-grain dofes, given at bed time, rubbed into a powder, with a little white fugar. If this quantity do not procure two or three motions in the courfe of the following day, the dofe may be increafed to three quarters of a grain, or even a grain. It may be repeated in this manner every night, or every fecond night, according to the degree of diftrefs and ftrength of the patient, until the ftools affume a natural appearance. This they feldom fail to do in a week or two, and then all griping and uneafinefs ceafe. It will rarely be neceffary to give more than from four to eight grains of calomel on fuch occafions occasions, and I can with the utmost confidence assert that I have never known it to do any mischief, and very seldom to fail of producing the desired effects. Nor is it any objection to the use of this medicine, that the patient labours under some degree of diarrhœa; the acrimony of the stools not unfrequently excites frequent and ineffectual efforts, which are to be removed only by a removal of the exciting cause. If absorbents possess any power in such cases, they owe it to their combination either with laxatives, essential oils, or distilled waters; and even with these aids I have commonly found them to afford but temporary ease.

That infants should be particularly liable to disorder in the biliary secretion can hardly appear surprising, when it is considered that in them the liver bears a much larger proportion to the weight of the body than in adults: This increased size of the liver, like that of the heart, probably diminishes gradually as the body advances towards maturity. The effects of this peculiarity, of structure of infants, are strongly manifested in most cases even after birth: although it be a common practice to keep their bowels discharging freely, and and though their ſtools be evidently loaded with bile, yet during the firſt week few infants eſcape ſome degree of jaundice (commonly called Yellow Gum.) Theſe facts prove a very copious ſecretion and excretion of bile at an early period, when the uncommon ſize of the liver is indiſputable; it appears to me probable that the ſame tendency to copious ſecretion muſt continue, in ſome degree, through infancy and childhood until this viſcus be reduced to its ordinary ſize. Medical practitioners have long remarked that well-prepared calomel agrees ſingularly well with the conſtitution of children in all thoſe diſeaſes wherein they have thought it prudent to employ it. Do not the peculiarities of ſtructure above noticed afford ſome explanation of this fact? The power of calomel in correcting green ſtools, and the uneaſineſs accompanying them, is a fact which I hope will be admitted by all practitioners who give it a fair trial.

Since the above remarks were written I have met with two or three caſes of violent bilious vomitings and cholics in infants, which were effectually relieved by much greater quantities of calomel than what I have ſtated above. To what extent it may be puſhed in very bad cafes my experience does not yet enable me to determine.

### *Of obftinate Coftivenefs.*

Every one, acquainted with the conftitution of infants, knows that it is natural to them to have three or four ftools every twenty-four hours; and that without fuch difcharges they feldom enjoy perfect health. A few inftances occur in practice where the inteftines of infants never difcharge their contents unlefs irritated by fome kind of phyfic. I have met with a good many fuch cafes, and after trying all the ordinary laxative medicines I could not fay that any of them was entitled to a preference. The effects of all were temporary only. An ingenious friend and correfpondent in London firft fuggefted to me a trial of calomel in fuch obftinate cafes. On his authority I have repeatedly had recourfe to it, and feldom without the beft effects. Whether it operates by promoting a flow of bile and other fecreted fluids into the inteftinal canal, on which the ftools of infants, from the nature of their food, muft very much depend; or by exciting, in a peculiar manner, the irritability of the nerves beſtowed on the inteſtines, I ſhall not pretend to decide.

*Of Convulſions in early Infancy.*

In the Tranſactions of this Academy * for the year 1789, I have given an account of the nine day fits, as they had been obſerved in the Lying-in Hoſpital of this city, and of certain modes of prevention which then appeared to have produced good effects, and which I am happy to add ſtill continue to do ſo.† Of methods of cure I was on that occaſion ſilent, becauſe no remedy had then been diſcovered even to retard the progreſs of that very fatal diſeaſe. About two years ago I was called in conſultation to a caſe of nine day fits, which

* See alſo Med. Facts and Obſ. Vol. III. p. 78.

† This aſſertion will be beſt underſtood by ſtating the following facts: Previous to the year 1782 the mortality of infants was one in ſix, or ſeventeen in the hundred. From 1782 'till 1788, a period of four years, it was one in nineteen, or from five to ſix in the hundred. During the laſt four years it has been nearly as one to twenty-ſix and a half, not altogether four in the hundred. See an abſtract of the regiſtry kept at the Lying in Hoſpital, by Mr. B. Higgins, (inſerted in the third volume of this work.)

appeared appeared hopelefs both to the attending phyfician and myfelf. As an experiment I propofed a grain of calomel to be given night and morning, knowing it to be well calculated to remove difeafe in the bowels, if fuch exifted, and a blifter to the fontanelle to relieve any fulnefs which might opprefs the brain. By the ufe of thefe remedies, and of tepid bathing, the infant recovered. As blifters and tepid bathing fo often fail in curing this difeafe, I was inclined to attribute much of our fuccefs to the calomel, and therefore I introduced the ufe of it in a fimilar manner into the Lying-in Hofpital; and although I cannot fay it actually cured one, yet it certainly afforded more obvious relief than any remedy hitherto tried. Several infants, whom the moft experienced nurfe-tenders apprehended to be ferioufly threatened, efcaped the difeafe; and it evidently mitigated the feverity of fymptoms in fome defperate cafes. Whether, in a pure atmofphere, and fituations otherwife more favourable for the exhibition of powerful remedies, it may be found to produce better effects, I propofe as a quere, to be determined by future obfervation? In the courfe of laft month I was fo fortunate in private practice as as to remove convulſions in an infant of three weeks old, by calomel and bliſtering, as above deſcribed, without the uſe of any other remedy.*

*Cutaneous Eruptions.*

It is a ſingular fact that infants on the breaſt are very ſubject to cutaneous eruptions of the herpetic kind, which in adults are commonly ſuppoſed to originate from acrimony in the fluids. It is not eaſy to conceive how acrimony is ſo frequently generated in the blood of infants, nouriſhed by the mildeſt of all fluids. It is a vulgar ſuppoſition that theſe diſeaſes are often occaſioned by improprieties in the diet and conduct of hireling nurſes; but they often happen to infants ſuckled by the mother, where no ſuſpicion of impropriety can be entertained: nay, the ſame tendency

* Since this paper was firſt read to the Academy, which is near two years ago, I think I have been ſtill more ſucceſsful in the treatment of the foregoing complaints, by adding to each doſe of calomel a grain or two of ſcammony or jalap, ſo as to render it more certainly and briſkly purgative. *June* 1795.

is obſerved in infants fed on ſpoon meat. I have been frequently tempted to think that nature intended ſuch eruptions to carry off ſurperfluous or redundant fluids from the conſtitution of infants; hence, perhaps, it is that they are moſt frequently to be met with among large infants, of a full habit. Viewing the ſubject in this light, one would be naturally inclined to encourage the diſcharge from ſuch eruptions; with me ſoap and water is a favourite application; by waſhing off filth, which obſtructs the pores, it allows a free exit both to perſpiration and to the diſcharge from the ulcerated parts; it alleviates the itching, which is not only very troubleſome, but abſolutely injurious to the infant's health, by interrupting its ſleep. All ointments and greaſy applications, having a contrary tendency to ſoap and water, ſeem to me objectionable. By diſcharging freely, the cutaneous complaints under conſideration often run their courſe with ſafety in a few weeks, and the patient is reſtored to health without the uſe of any internal medicine. When, however, the general health ſeems to be impaired, and the violence of ſymptoms renders medicine neceſſary, I have not found any remedy to be put in competition tion with calomel, given in the ſame manner as for correcting griping and green ſtools.

Although not immediately connected with my preſent ſubject, it may not be altogether uſeleſs to remark, that along with an herpetic eruption on infants there is ſometimes a mixture of itch which creates a very troubleſome diſeaſe, and one very liable to be miſunderſtood. It is principally by appearances on the nurſe that the exiſtence of itch on the infant can be diſcovered. Herpes on the infant we know generally brings out ſome eruption on the nurſe, but on accurate examination this will be found very different in its progreſs and effects from itch. The mode of curing itch in adults, when diſcovered, is now well aſcertained; but there is great reaſon to doubt whether the ſame treatment be ſafely applicable to infants, and eſpecially when itch is combined with herpes. Coughs of a very dangerous tendency have been obſerved to ariſe after the application of ſulphur ointment to infants. Fortunately there is no abſolute neceſſity for its uſe. Common ſtick brimſtone, bruiſed and boiled for ſome time in water, gives a ſulphureous impregnation, which uſed as a tepid bath every night at bed time ſeldom fails to cure the itch in infants in a few weeks, without any riſk of obſtructing the pores of the ſkin, or of repreſſing too much the herpetic eruption. In all ſuch caſes it is prudent to make the nurſe rub the eruption on her with ſulphur ointment, while at the ſame time ſhe takes flour of ſulphur and magneſia internally, ſo as to keep the bowels moderately open. The infant ſhould alſo ſleep in the ſame bed with the nurſe during this operation.

I have thus ſketched, in a haſty manner, the outline of what accident and ſome reflection have ſuggeſted to me on a few ſubjects, in my opinion not perfectly underſtood. To thoſe who know me, hurry of buſineſs (in an irregular and fatiguing profeſſion) will apologize for many defects. The points in doubt regard a numerous claſs of the innocent and helpleſs in the community, who, when ſick, are too frequently entruſted to the care of prejudiced old women, or of men not much better qualified to practice phyſic. I, therefore entreat gentlemen to enter diſpaſſionately into the inveſtigation of the doubts here ſubmitted to their conſideration. The alleviation of pain, and the prolongation of human life, at a period of its greatest frailty, are objects surely not beneath the notice of any man; and as I pretend not to infallibility, the reader may be assured I feel as much interested on this occasion to have *error detected* as to have *truth confirmed.*

